# Interleukin-17 receptor A (IL-17RA) as a central regulator of the protective immune response against *Giardia*

**DOI:** 10.1038/s41598-017-08590-x

**Published:** 2017-08-17

**Authors:** Oonagh Paerewijck, Brecht Maertens, Leentje Dreesen, Frederik Van Meulder, Iris Peelaers, Dariusz Ratman, Robert W. Li, Erik Lubberts, Karolien De Bosscher, Peter Geldhof

**Affiliations:** 1 Department of Virology, Parasitology and Immunology, Laboratory of Parasitology, Faculty of Veterinary Medicine, Ghent University, Merelbeke, Belgium; 2 VIB Department of Medical Protein Research, Receptor Research laboratories, Nuclear Receptor Lab, Faculty of Medicine and Health Sciences, Ghent University, Ghent, Belgium; 3 United States Department of Agriculture, Agriculture Research Service, Animal Genomics and Improvement Laboratory, Beltsville, Maryland United States of America; 4 Departments of Rheumatology and Immunology, Erasmus MC, University Medical Center, Rotterdam, The Netherlands

## Abstract

The protozoan parasite *Giardia* is a highly prevalent intestinal pathogen with a wide host range. Data obtained in mice, cattle and humans revealed the importance of IL-17A in the development of a protective immune response against *Giardia*. The aim of this study was to further unravel the protective effector mechanisms triggered by IL-17A following *G*. *muris* infection in mice, by an RNA-sequencing approach. C57BL/6 WT and C57BL/6 IL-17RA KO mice were orally infected with *G*. *muris* cysts. Three weeks post infection, intestinal tissue samples were collected for RNA-sequencing, with samples from uninfected C57BL/6 WT and C57BL/6 IL-17RA KO animals serving as negative controls. Differential expression analysis showed that *G*. *muris* infection evoked the transcriptional upregulation of a wide array of genes, mainly in animals with competent IL-17RA signaling. IL-17RA signaling induced the production of various antimicrobial peptides, such as angiogenin 4 and α- and β-defensins and regulated complement activation through mannose-binding lectin 2. The expression of the receptor that regulates the secretion of IgA into the intestinal lumen, the polymeric immunoglobulin receptor, was also dependent on IL-17RA signaling. Interestingly, the transcriptome data showed for the first time the involvement of the circadian clock in the host response following *Giardia* infection.

## Introduction

The intestinal protozoan parasite *Giardia duodenalis* has a wide vertebrate host range with high prevalences found both in production and companion animals. In humans, infections reach up to 280 million each year^1^. *Giardia* has a simple and direct life cycle, presenting itself in 2 morphological stages, i.e. the cyst and the trophozoite stage. Cysts are able to withstand the harsh environmental conditions outside the host, whereas trophozoites are adapted to the specific conditions in the intestine of the host. After the ingestion of infective cysts, which are present in the environment, such as in contaminated water or food, excystation occurs inside the host. This leads to the release of two flagellated trophozoites from each cyst. These trophozoites reside in the small intestine, where they attach to the mucosa and the surface of the epithelium without invading the tissue^2^. An infection with *Giardia* can pass without any clear symptoms, but can also lead to the clinical condition of giardiasis, which is characterized by gastro-intestinal complaints such as diarrhoea, abdominal pain, nausea and weight loss. While in most hosts these symptoms rapidly disappear, in others a chronic situation develops, which can last for several months^3^.

In recent years, several studies have highlighted the importance of IL-17A in orchestrating the protective immune response against *Giardia*. First, Solaymani-Mohamaddi and Singer showed *in vitro* production of IL-17 by spleen cells after stimulation with *Giardia* extract^4^. Furthermore, an *in vivo* study in calves demonstrated a strong proliferation of IL-17-producing CD4^+^ αβ T-cells starting from 5 weeks post infection^5^. Similarly, Saghaug *et al*. showed that a *G*. *duodenalis* infection in humans also evoked an IL-17A-producing memory CD4^+^ T cell response^6^. Finally, infections in mice with their natural parasite *G*. *muris* ^7, 8^ or with *G*. *duodenalis* ^8^ provoked a strong upregulation of IL-17A starting from week 1 post infection onwards. Importantly, mice deficient in either IL-17A or IL-17 receptor A (IL-17RA) were unable to clear a *Giardia* infection in comparison to wild type mice^7, 8^.

The functional role of IL-17A in the intestine can be very broad and has been described as being dual with both protective and pathological effects. IL-17A can influence many different cell types, such as T cells, B cells, macrophages, neutrophils and epithelial cells to produce various factors such as cytokines, chemokines, antimicrobial peptides, mucins and tight junction proteins^9^. Pathological, pro-inflammatory roles are mainly seen in autoimmune and inflammatory disorders, yet it is increasingly clear that IL-17A plays an important role in intestinal immune homeostasis and host defense mechanisms. However, insights in the precise protective effector mechanism(s) triggered by IL-17A following a *Giardia* infection are still scarce. Dann *et al*.^8^ previously reported on the outcome of a microarray analysis performed to identify genes differentially expressed between C57BL/6 WT and IL-17RA KO mice 2 weeks after a *G*. *muris* infection. This resulted in the identification of 6 genes that were preferentially induced in the C57BL/6 WT mice and not in the IL-17RA KO mice, including resistin-like molecule β (Retnlb), β-defensin 1 (Defb1), serum amyloid A1 and A2 (Saa1 and Saa2), a putative TNF-resistance related protein (U90926) and a phospholipase A2 (Pla2g4c)^8^. The authors also reported a decreased mucosal IgA secretion in IL-17A^−/−^ mice. In order to further extend our knowledge on the IL-17A-induced anti-*Giardia* immune response, in the current study RNA deep sequencing technology was applied as an unbiased way to analyse the intestinal response following a *G*. *muris* infection in both C57BL/6 WT and C57BL/6 IL-17RA KO mice. The outcome of this analysis indicated that a *Giardia* infection triggered an IL-17RA-dependent transcriptional upregulation of a wide array of antimicrobial proteins and complement factors, which, in combination with an intestinal IgA response seems to be important to confer protection against this parasite.

## Materials and Methods

### Infection experiments and tissue sample collection

All animal experiments were conducted in accordance with the E.U. Animal Welfare Directives and VICH Guidelines for Good Clinical Practice. Ethical approval to conduct the studies was obtained from the Ethical Committee of the Faculty of Veterinary Medicine, Ghent University (ethical committee approval numbers EC 2012/027 and EC 2012/176).

In all infection experiments, mice were orally infected with 10^3^ *G*. *muris* cysts suspended in 0.2 mL phospahte-buffered saline (PBS) and uninfected control groups were included.

Infection experiment 1 was previously described by Dreesen *et al*.^7^. In summary, this study consisted of 2 groups of 10 female C57BL/6 IL-17RA KO and 10 female C57BL/6 WT mice of 6 weeks old at the start of the experiment. Five mice of each group were orally infected with *G*. *muris* cysts. The five remaining mice of each group served as uninfected negative controls. Cyst counts were performed every 2 days to monitor the course of infection. All mice were sacrificed 21 days post infection (p.i.) and intestinal tissue samples were collected and processed as previously described^7^. The samples collected during this experiment were subjected to RNA sequencing.

For additional qRT-PCR analyses, small intestinal samples from another experiment described by Dreesen *et al*.^7^ were used. Briefly, 6 week-old female C57BL/6 mice were infected with *G*. *muris* cysts, and uninfected control groups of mice were included. Mice were sacrificed at day 7, day 14 and day 21 p.i., and small intestinal tissue was collected. The number of mice was 5 in each group for each timepoint^7^.

For further analysis of circadian clock-associated genes, a third infection experiment was performed in which 5 female C57BL/6 WT mice were likewise infected with *G*. *muris* cysts and 5 female C57BL/6 WT mice served as uninfected controls. The mice were sacrificed 21 days p.i., at 1 pm, and intestinal tissue samples were collected and processed as previously described^7^.

Infection experiment 4 involved a *G*. *muris* infection in B6.129S4-Mbl1^tm1Kata^ Mbl2^tm1Kata^/J mice on a C57BL/6 background (C57BL/6 Mbl KO) (Jackson Laboratories) and their respective C57BL/6 WT controls. C57BL/6 Mbl KO and C57BL/6 WT mice were housed together in the same cage. Ten female C57BL/6 WT and 10 C57BL/6 Mbl KO mice were infected with 10^3^ *G*. *muris* cysts. Cyst counts were monitored daily, starting from day 1 p.i., as previously described^7^. Five animals of each group were sacrificed at day 7 p.i. and the remaining animals at day 21 p.i. Intestinal tissue was collected and the trophozoites present in the intestine counted. Hereto, the small intestine was removed, starting from 2 cm distal to the gastrointestinal junction. The segment was placed in PBS and incubated on ice for 20 minutes, before counting the trophozoites by using a hemacytometer. The counts are expressed as the absolute number of trophozoites present in the small intestine. In addition, a duodenal tissue sample of 2 cm long, distal to the gastrointestinal junction, was taken from each animal. The samples were snap-frozen in liquid nitrogen and subsequently used for the extraction of total RNA, as previously described^7^.

### RNA sequencing and analysis

Total RNA from experiment 1 (RNA integrity number (RIN) >8.5) was processed using an Illumina TruSeq RNA sample prep kit following the manufacturer’s instructions (Illumina, San Diego, CA, USA). Individually barcoded libraries were then pooled at an equal molar ratio and sequenced at 2 × 50 bp/read using an Illumina HiSeq. 2000 sequencer, as described previously^10, 11^. Approximately 31 million paired-end sequence reads per sample (mean ± SD = 31.3507 ± 5.3253 million; N = 20) were generated. The quality of the samples was assessed via FastQC software. After inspection 2 pre-processing steps were performed using custom python scripts: (i) end trimming: 3 bp’s from 5′ site and 1 bp from 3′ site (lower quality), (ii) elimination of reads with mean quality score <31 and total number of non-identified bases (N’s) >2. The obtained reads, varying between 50 mio and 80 mio reads per sample, were then mapped to the mouse mm10 genome assembly using Tophat (v2.0.11). Count tables were created with htseq-count (v 0.6.0) software using UCSC mm10 annotation and were used as an input for the analysis using the DESeq. 2 R package. Differential expression analysis was performed, based on the following 4 major comparisons: C57BL/6 WT infected versus C57BL/6 WT uninfected control, C57BL/6 IL-17RA KO infected versus C57BL/6 IL-17RA KO uninfected control, C57BL/6 WT uninfected control versus C57BL/6 IL-17RA KO uninfected control and C57BL/6 WT infected versus C57BL/6 IL-17RA KO infected. The filters that were used to identify differential expression were an adjusted p-value lower than 0.05 and an absolute log2 fold change greater than 1. The four comparisons where then further subdivided into 15 exhaustive categories and a Venn diagram was created (Venny 2.1 software). Ingenuity Pathway Analysis software was used to link genes in the dataset to particular biological functions and pathways. Heatmaps were drawn using the gplots R package and show scaled expression values clustered based on Euclidean distance.

### Real-time quantitative PCR

A quantitative Real-Time PCR (qRT-PCR) approach was used to measure the relative mRNA expression levels of several genes in the small intestine. Samples obtained at day 7, day 14 and day 21 p.i. from C57BL/6*G*. *muris* infected mice and from C57BL/6 uninfected control mice, as well as at day 21 p.i. from Mbl KO *G*. *muris* infected mice and Mbl KO uninfected control mice, were used. The sequences of all the primers that were used can be found in supplementary Table 1. QRT-PCR and normalization of the data, which was based on the housekeeping genes Hprt1 and Tbp, was essentially performed as previously described^7^. Gene transcription levels were evaluated based on fold between the different groups. Statistical analysis was carried out using GraphPad Prism software. A one-way ANOVA followed by a Dunn’s multiple comparison test was used to determine differences between the different groups. A P-value ≤ 0.05 was considered significant.

### Histology

The presence of angiogenin 4 (Ang4) protein was visualised in intestinal tissue. Hereto, small intestinal tissue obtained from C57BL/6 WT *G*. *muris* infected mice was stained with a polyclonal sheep anti-mouse-Ang4 antibody, kindly provided by Dr. R. Forman, using a general staining protocol for paraffin sections. Briefly, 4 μm thin sections of formalin-fixed, paraffin-embedded tissues were cut and placed onto 3-aminopropyltriethoxysilane coated glass slides. The sections were deparaffinised and subsequently blocked with a 4% bovine serum albumin solution in PBS, followed by incubation with the primary anti-Ang4 antibody (12 μg/ml). Next, an incubation step with a biotin labeled secondary donkey anti-sheep IgG antibody (SantaCruz) was performed, followed by DAB staining by means of a Vectastain Elite ABC kit (Vector Laboratories). A negative antibody control was included, in which sections were incubated with the secondary donkey anti-sheep IgG antibody after blocking and DAB stained.

### *In vitro* incubation of *G*. *duodenalis* trophozoites with angiogenin 4

Plasmid containing the murine Ang4 coding sequence (pET3a-Ang4) (kindly provided by Prof. Simon Carding and Dr. Isabelle Hautefort), was introduced into a pPICZalfaB expression vector, followed by transformation into electrocompetent Top10F-cells. Plasmid DNA was purified from pPICZalfaB Top10F-cells with QIAfilter Plasmid Purification Kit (Qiagen) and subsequently linearised. Linear plasmid DNA was transformed into KM071H-cells, which were grown by fermentation in BMGY- and BMMY-medium. Expressed protein was isolated by collecting, filtrating and concentrating culture supernatant and purified by cation exchange on a Resource S column (Sigma Aldrich). *G*. *duodenalis* trophozoites, human assemblage B isolate GS, were kept in axenic culture in modified TYI-S-33 medium^12^. *G*. *duodenalis* trophozoites were incubated with the Ang4 recombinant protein in 96-well plates. Trophozoite numbers ranged from 1 × 10^4^ to 5 × 10^5^ per well. They were incubated with Ang4 protein for 2 h, 8 h, 16 h and 24 h. The concentration of Ang4 ranged from 0,5 to 100 μM in 300 of μL of *Giardia* TYI-S-33 medium. Fenbendazole 0,5 μM (Panacur, MSD) served as a positive control for the killing of trophozoites. After the respective time points, TYI-S-33 medium was removed and replaced by PBS. Finally, 2 μg of resazurin (Sigma-Aldrich) was added per well, as described by Bénéré *et al*.^13^. This blue dye is converted into fluorescent pink by metabolically active trophozoites. After 8 h, fluorescence was measured with a Tecan SpectraFluor fluorescence reader (λ_ex_ 492 nm–λ_em_ 595 nm).

### Western blot

The presence of Mbl2 protein in the small intestinal tissue of C57BL/6 WT *G*. *muris* infected mice and C57BL/6 WT uninfected control mice, sampled at 21 days p.i., was measured by means of Western blotting. Hereto, the tissue was ground into powder in liquid nitrogen. A water-soluble extract was prepared by dissolving the powder in 150 mM PBS. After centrifugation at 16 000 rpm for 15 minutes, the supernatant was frozen at −80 °C until further use. The pellet was subsequently solubilised in PBS with a 1% protease inhibitor cocktail (Sigma-Aldrich) and with 1% Triton X-100, centrifuged at 16 000 rpm for 1 hour and the supernatant collected and frozen at −80 °C. Protein content of the supernatant was measured with the Pierce BCA protein assay (Thermo Scientific). Two 15% SDS-PAGE gels were run with 30 μg of protein and 1 gel was stained with SimplyBlue SafeStain (Thermo Scientific), to confirm equal loading and concentration. The second gel was blotted onto a nitrocellulose membrane. Subsequently, the blot was washed and incubated with blocking solution (PBS with 0,5% Tween80), followed by an incubation for 24 h at 4 °C with primary rat anti-Mbl2 antibody (Abcam), diluted 1/50 in blocking solution. Following washing, the blot was incubated for 1 h with secondary goat anti-rat-HRP (SantaCruz), diluted 1/1000 in blocking solution. Mbl2 protein was visualised by incubation with DAB dissolved in Tris-buffered saline pH 7.6 (Sigma-Aldrich).

### ELISA

Fecal IgA antibody levels were measured by enzyme-linked immunosorbent assays (ELISA). Total IgA levels were measured in fecal samples from WT and Mbl KO uninfected control mice. *Giardia*-specific IgA levels were measured in fecal samples from WT infected and uninfected control mice. Fecal samples were dissolved in PBS with 0,01% sodium azide and 1% protease inhibitor cocktail (Sigma-Aldrich). Proteins were extracted by vortexing and centrifuging at 16 000 g for 10 min. Supernatants were stored at −80 °C until further use. For measurement of total IgA, Maxisorp 96-well plates (Nunc) were coated with 2 μg/ml sheep anti-mouse IgA (Sigma-Aldrich), for 16 h at 4 °C. For measurement of *Giardia*-specific IgA levels, plates were coated with *G*. *duodenalis* trophozoites. Hereto, trophozoites that were kept in axenic culture in modified TYI-S-33 medium, were centrifuged for 5 min at 800 g, resuspended and washed 3 times in PBS. Next, they were resuspended in 8% formaldehyde solution. 2 mL of formaldehyde solution was added to trophozoites derived from 10 mL of culture. 100 μl of this solution was added to each well of the plates, which were pre-incubated with poly-L-lysine (Sigma-Aldrich). Trophozoites were allowed to attach to the plates for 48 hours and the plates were subsequently dried at 37 °C for 24 hours. After washing with PBS-Tween 20 (0,05%) solution (PBST), all plates were blocked with 2% bovine serum albumin (BSA) in PBST. After blocking, the plates were incubated for 1 hour at room temperature with the fecal extracts in a 1/5 dilution. For measurement of total and *Giardia*-specific IgA levels, the plates were washed and incubated for 1 hour at room temperature with goat anti-mouse IgA-HRP (Sigma-Aldrich) in a 1/1000 dilution in PBST.

Binding of recombinant Mbl2 to *Giardia* trophozoites was also measured by ELISA. Hereto, plates were coated with trophozoites as described above. In a first step, recombinant Mbl2 (R&D Systems) was added to the plates in different concentrations, ranging from 0.02–4 μg/ml and incubated for 2 hours at room temperature. Plates were washed 3 times and incubated for 1 hour with rat anti-Mbl2-antibody (2 μg/ml) (Abcam). After washing, plates were finally incubated for 1 hour with goat anti-rat-HRP (SantaCruz Biotechnologies) in a 1/500 dilution in PBST. In a second step, trophozoite-coated plates were incubated for 1 hour at room temperature with fecal samples of WT infected and uninfected control mice, in a 1/5 dilution. After washing, 2 μg/mL of recombinant Mbl2 (R&D systems) was added to each well and the binding of Mbl2 to the trophozoites was subsequently measured as described above.

All reactions were visualised with ABTS in ABTS buffer (Roche). Optical density was measured at 405 nm with a Tecan plate reader, diminished by conjugate control levels measured at 492 nm.

## Results and Discussion

### Transcriptional changes in C57BL/6 wild type mice following a *G*. *muris* infection

**Figure 1:**
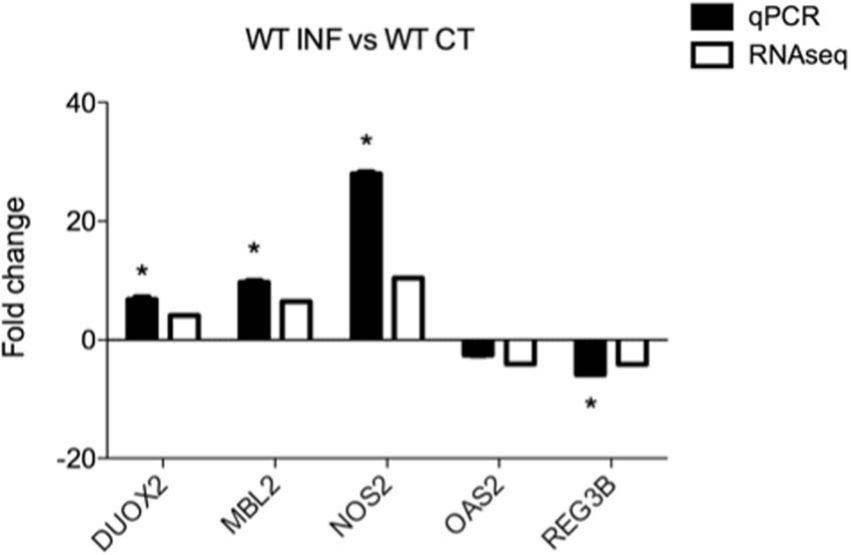
qRT-PCR analysis for genes identified by RNA-seq as differentially expressed between *G*. *muris* infected and uninfected C57BL/6 WT mice. Genes that are significantly differentially regulated between the 2 groups, as measured by qRT-PCR, are marked with an asterisk (*p < 0.05).

Differential expression analysis performed on the sequence reads obtained from C57BL/6 WT infected versus C57BL/6 WT uninfected control mice resulted in the identification of 844 differentially expressed (DE) genes. Of these, 483 genes were upregulated and 361 genes downregulated in the intestinal tissue of infected mice in comparison to uninfected controls (Table S2). In order to validate the RNA-seq data, qRT-PCRs assays were performed for 6 DE genes, including angiogenin 4 (Ang4), dual oxidase 2 (Duox2), mannose binding lectin 2 (Mbl2), nitric oxide synthase 2 (Nos2), 2′-5′-oligoadenylate synthetase 2 (Oas2) and regenerating islet-derived 3 beta (Reg3B). Consistent with the RNA-seq analysis, an upregulation could be observed for Mbl2, Duox2 and Nos2 and a downregulation for Oas2 and Reg3B (Fig. 1). Ingenuity Pathway Analysis (IPA), performed on the 844 DE genes, revealed an increase in the activity of functions mainly related to the development and repair of tissue (Table S3). The genes involved in most of the top 5 impacted and increased functions were IL-17A, Nos2, T-cell acute lymphocytic leukemia 1 (Tal1), the transcription factor GATA binding protein 2 (Gata2) and tumor necrosis factor superfamily member 15 (Tnfsf15).

**Figure 2:**
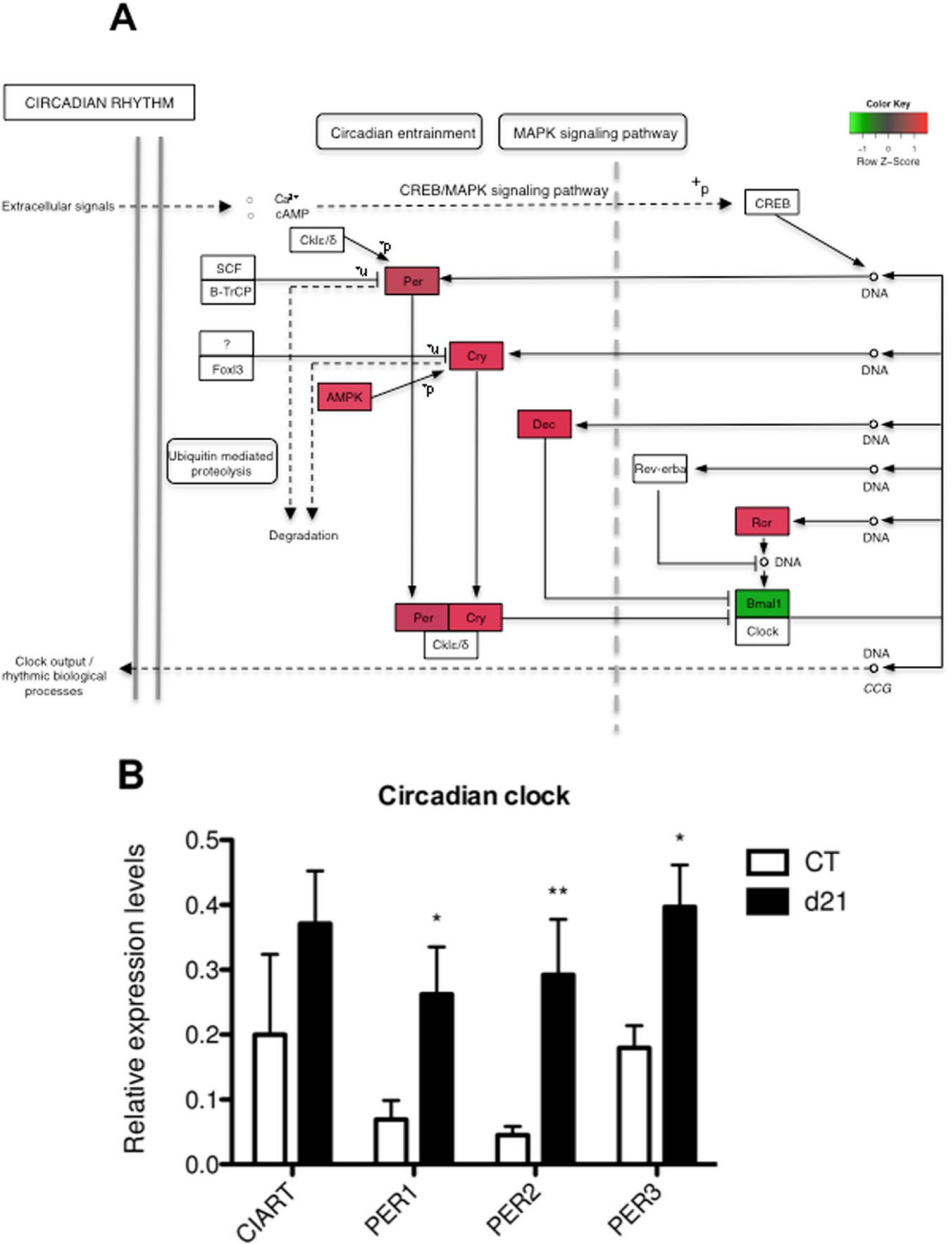
*G*. *muris* infection in C57BL/6 WT mice has an impact on the circadian clock. (**A**) Kyoto Encyclopedia of Genes and Genomes (KEGG) pathway for the circadian clock (©Kanehisa Laboratories)^50^. Red and green respectively indicates transcriptional up- or down-regulation in C57BL/6 WT infected mice compared to C57BL/6 WT uninfected control mice, as identified by RNA-seq. (**B**) Relative mRNA expression levels of the circadian clock associated genes Ciart, Per1, Per2 and Per3 in *G*. *muris* infected C57BL/6 WT mice as measured by qRT-PCR at day 21 post infection compared to uninfected control mice (*p < 0.05, **p < 0.01).

Interestingly, among the 15 highest upregulated genes in C57BL/6 WT infected mice (Table 1), 3 genes were found that are associated with the circadian or day-night rhythm, i.e. Per3, Per2, and Ciart. An additional three genes involved in this pathway were also identified as being upregulated following infection, i.e. Per1, Cry2 and Bhlhe41. All these genes encode for inhibitors of the transcription factors Bmal1 and Clock, which are the central regulators of the circadian clock. CLOCK and BMAL1 will heterodimerize in the cytoplasm to form a complex that, following translocation to the nucleus, initiates transcription of a range of target genes, including their own inhibitors Per1, Per2, Per3, Cry2, Bhlhe41 and Ciart, creating a negative feedback loop^14^. In accordance with the transcriptional upregulation of these inhibitors following a *Giardia* infection, the RNAseq data also indicated that the transcription of Bmal1 was downregulated in C57BL/6 WT infected mice compared to C57BL/6 WT uninfected controls (Fig. 2A). Because the transcription levels of genes associated with circadian rhythm vary substantially depending on the time of the day, an additional infection study was performed in which C57BL/6 WT infected mice and C57BL/6 WT uninfected control mice were sacrificed at the exact same time of day, i.e. 1 pm. Consistent with the first study, a significant upregulation was again observed for Per1, Per2 and Per3 at 21 days post infection in infected mice compared to uninfected control animals (Fig. 2B). The functional implications of the transcriptional changes of clock genes following *Giardia* infection are still unclear. Infection experiments in mice deficient for genes associated with the circadian rhythm, such as Clock, Per2, Bmal1 and Cry, have shown an effect of these genes on the response to bacterial challenge, as reviewed by Tsoumtsa *et al*.^15^. The time of day during which organisms are infected with certain microbials also seems to influence the extensiveness of the infection and the associated antimicrobial response, as is the case for a *Salmonella* Typhimurium infection in mice^16^. In addition, it has been shown that expression of clock genes in the intestinal tissue controls gastrointestinal functions such as motility, cell proliferation and migration^17^. It is therefore possible that this response is part of a defence mechanism in an attempt to remove the parasite from the intestinal lumen.

**Table 1:** Top 15 most upregulated genes as identified by RNA-seq in C57BL/6 WT infected mice versus C57BL/6 WT uninfected control mice.

Gene symbol	Gene name	RefSeq ID	Fold change
PER3	Period circadian clock 3	NM_011067	13,49
LY6G6C	Lymphocyte antigen 6 complex, locus G6C	NM_023463	12,65
HLF	Hepatic leukemia factor	NM_172563	12,33
DEFB1	Defensin beta 1	NM_007843	11,79
NOS2	Nitric oxide synthase 2, inducible	NM_010927	10,42
IL17A	Interleukin 17A	NM_010552	9,67
PER2	Period circadian clock 2	NM_011066	9,35
CIART	Circadian associated repressor of transcription	NM_001033302	9,33
SLC10A2	Solute carrier family 10, member 2	NM_011388	8,59
GM15299	Predicted pseudogene 15299	NM_001170955	7,89
MRGPRA9	MAS-related GPR, member A9	NM_001288801	7,77
CEMIP	Cell migration inducing protein	NM_030728	7,36
ANG4	Angiogenin, ribonuclease A family, member 4	NM_177544	7,20
RGS18	Regulator of G-protein signaling 18	NM_022881	7,09

**Figure 3:**
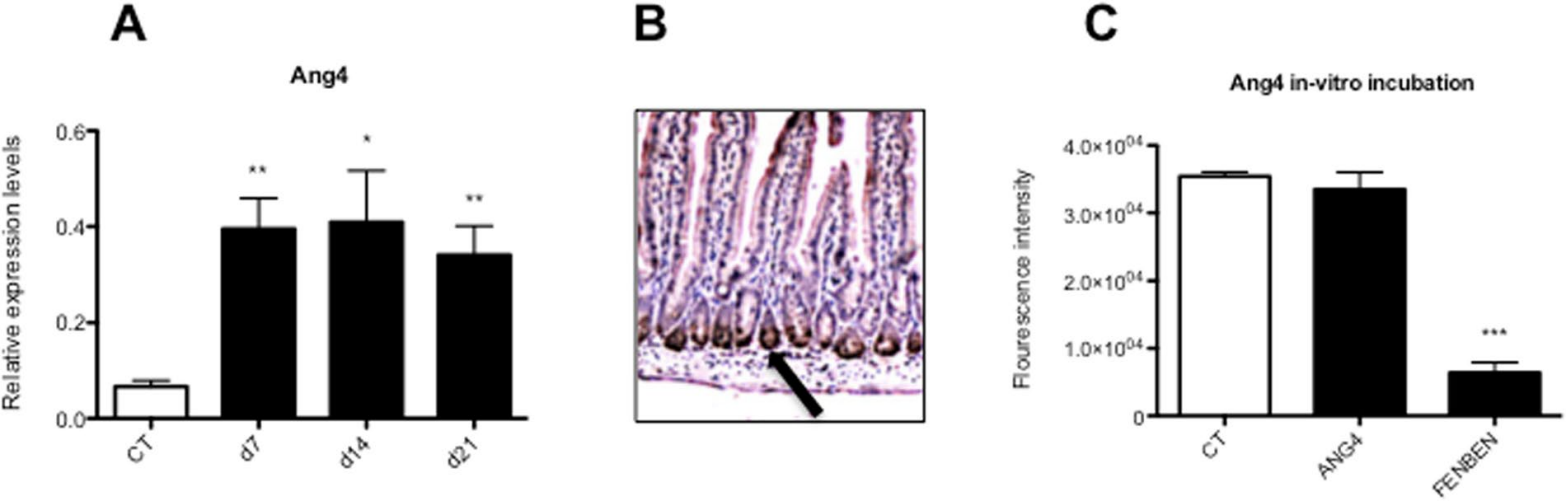
Ang4 is transcriptionally upregulated in paneth cells in the small intestine of mice following an infection with *G*. *muris* but has no direct effect on trophozoite viability. (**A**) Relative mRNA expression levels of Ang4 in the small intestine of C57BL/6 WT *G*. *muris* infected mice versus C57BL/6 WT uninfected controls, as measured by qRT-PCR. Expression levels were measured at day 7, day 14 and day 21 post infection (*p < 0.05). (**B**) Histological analysis with an anti-Ang4 antibody revealed the presence of Ang4 protein in paneth cells (indicated by black arrow) in the small intestine of C57BL/6 WT infected mice. (**C**). *In vitro* incubation of *G*. *muris* trophozoites with angiogenin 4 recombinant protein (100 μM) and fenbendazole (0.5 μM) as a positive control. Metabolic activity of trophozoites was measured as fluorescence intensity of the reduced colorimetric agent resazurin (***p < 0.001).

Another interesting gene amongst the top 15 highest upregulated genes in C57BL/6 WT mice was angiogenin 4 (Ang4). Although Ang4 was originally identified as a factor that induces neovascularization, it is also known as an important antimicrobial protein produced by Paneth cells^18^. Experimental infections of mice with the gastrointestinal nematode *Trichuris muris* revealed a positive correlation between Ang4 expression and expulsion of the parasite^19, 20^, suggesting a potential anti-parasitic function. Interestingly, Ang4 was also identified in the microarray study of Tako *et al*.^21^ as being 4-fold upregulated in the intestine of mice 10 days after a *G*. *duodenalis* infection. In order to further investigate the role of Ang4 following a *G*. *muris* infection, the transcriptional response of this gene was analysed at different time points following infection by qRT-PCR. The outcome of this analysis indicated that a significant transcriptional upregulation was already observed from week 1 post infection onwards (Fig. 3A). Furthermore, histological analysis revealed the presence of Ang4 in the small intestinal paneth cells of *G*. *muris* infected mice (Fig. 3B). Finally, in order to assess whether Ang4 had a direct effect on the viability of *Giardia*, trophozoites of *G*. *duodenalis* were incubated *in vitro* with different concentrations of a recombinantly produced Ang4. As shown in Fig. 3C the presence of Ang4 in *G*. *duodenalis* trophozoite cultures, up to a concentration of 100 μM, appeared to have no direct effect on their viability, this in contrast to fenbendazole which was used as a positive control. The reasons for the lack of a direct effect of Ang4 on trophozoites are currently unclear. In bacteria, Ang4 is capable of impairing membrane integrity to exert a direct effect^22^. However, Hooper e*t al*. have also demonstrated that although Ang4 has microbicidal activity against certain bacteria, other bacteria appeared to be resistant. This shows that species-specific features can influence the susceptibility to Ang4^18^.

**Figure 4:**
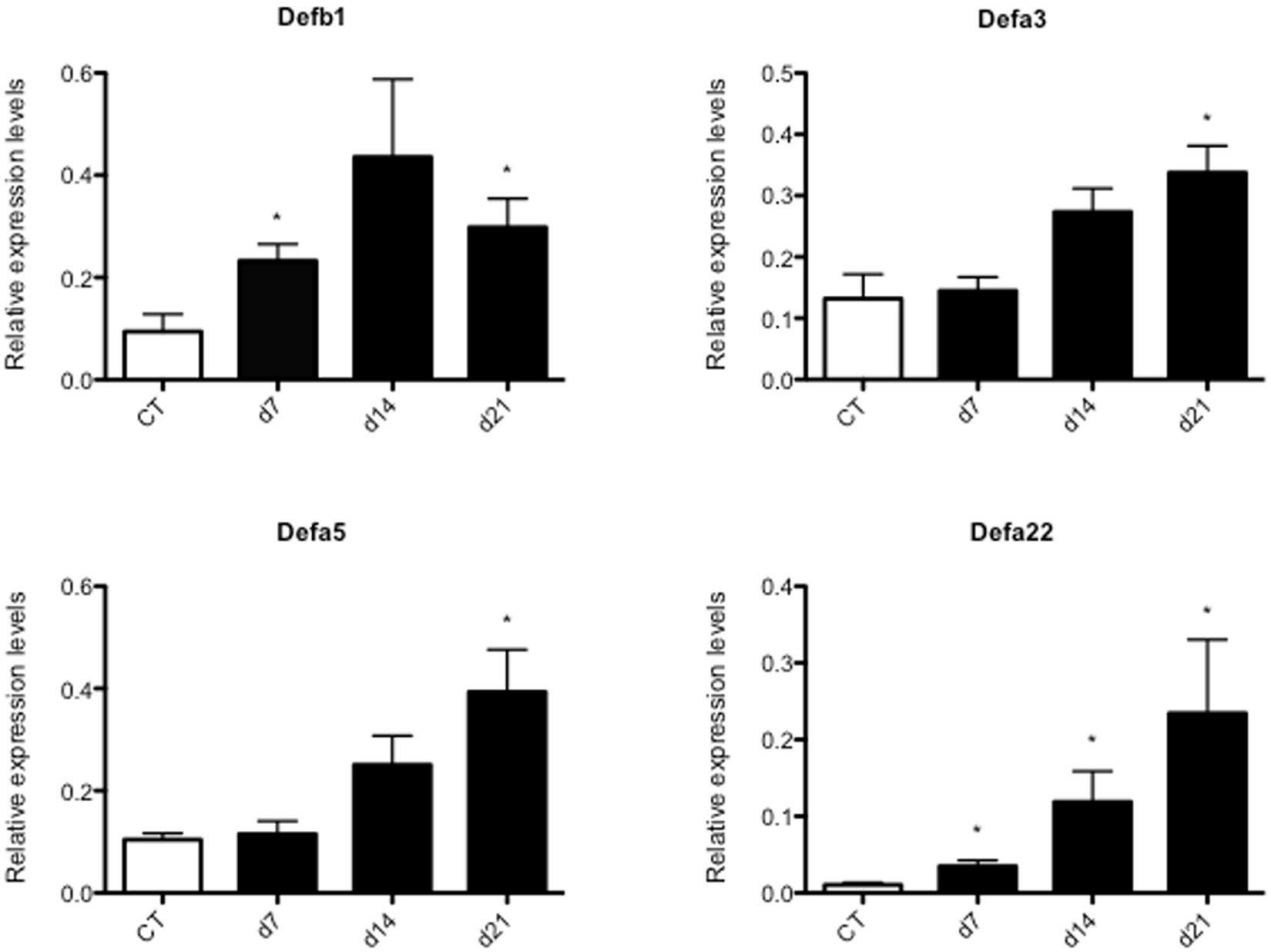
Alpha- and beta-defensins are transcriptionally upregulated in the intestine of C57BL/6 WT mice following *G*. *muris* infection. Relative mRNA expression levels of Defb1, Defa3, Defa5 and Defa22 in C57BL/6 WT *G*. *muris* infected mice were measured by qRT-PCR at day 7, 14 and 21 post infection, compared to C57BL/6 WT uninfected controls (*p < 0.05).

Next to Ang4, a large number of additional antimicrobial proteins were also transcriptionally upregulated following infection, including α- and β-defensins, lectins and phospholipases (summarized in Table 2), with β-defensin 1 (Defb1) being the most upregulated. The upregulation of Defb1 and 3 α-defensins (Defa3, Defa5 and Defa22) at day 21 after *G*. *muris* infection was confirmed by qPCR analysis. This analysis also revealed a small upregulation at day 7 and day 14 p.i. (Fig. 4). Although Defb1 was also identified as being upregulated in the microarray study of Dann *et al*.^8^, its role in the anti-*Giardia* immune response is still unclear. It is also noteworthy that matrix metalloprotease 7 (Mmp7), which is responsible for the activation of α-defensins through the proteolytic removal of the inhibitory pro-peptide^23^, was also significantly upregulated in infected mice. Mmp7 was also one of the induced transcripts in the microarray study of Tako *et al*. 10 days post a *G*. *duodenalis* infection in mice. They further showed that mice homozygous for a deletion of the Mmp7 gene were unable to fully eliminate *G*. *duodenalis* ^21^. On the other hand, Eckmann reported that Mmp7-deficient mice were fully able to control and eliminate a *G*. *muris* infection^24^. Further research is therefore necessary to fully understand the role of Mmp7, and by extension the α-defensins, in the protective immune response against *Giardia*.

**Table 2:** List of upregulated antimicrobial genes as identified by RNA-seq in C57BL/6 WT infected mice versus C57BL/6 WT uninfected control mice.

Gene symbol	Gene name	RefSeq ID	Fold change
DEFB1	Defensin beta 1	NM_007843	11,79
NOS2	Nitric oxide synthase 2, inducible	NM_010927	10,42
DEFA-PS1	Defensin, alpha, pseudogene 1	NR_003146	5,89
DEFA22	Defensin, alpha, 22	NM_207658	4,92
DEFA21	Defensin, alpha, 21	NM_183253	4,54
GSDMA2	Gasdermin A2	NM_029727	4,52
GSDMC	Gasdermin C	NM_031378	3,90
GSDMC2	Gasdermin C2	NM_177912	3,75
DEFA3	Defensin, alpha, 3	NM_007850	3,44
DEFA5	Defensin, alpha, 5	NM_007851	3,37
DEFA17	Defensin, alpha, 17	NM_001167790	3,32
DEFA26	Defensin, alpha, 26	NM_001079933	3,14
REG4	Regenerating, islet-derived family, member 4	NM_026328	2,77
ITLN1	Intelectin 1	NM_010584	2,72
DEFA-RS1	Defensin, alpha, related sequence 1	NM_007844	2,47
PLA2G4F	Phospholipase A2, group IVF	NM_001024145	2,44
PLA2G2A	Phospholipase A2, group IIA	NR_002926	2,40
PLA2G2F	Phospholipase A2, group IIF	NM_012045	2,23
DEFA24	Defensin, alpha, 24	NM_001024225	2,22
RETNLG	Resistin like gamma	NM_181596	2,16
SLPI	Secretory leukocyte peptidase inhibitor	NM_011414	2,01

Another group of genes that was identified in the study of Tako *et al*.^21^ as being highly upregulated following *G*. *duodenalis* infection, were genes linked to mast cell responses. Mast cells have previously been shown to play a role in the immune response against *G*. *duodenalis* ^25, 26^ and *G*. *muris* ^27^ in several studies. In contrast, in our current dataset genes linked to mast cell responses were not impacted. This could be due to the different timepoints of analysis, e.g. 10 days p.i. in the study of Tako *et al*.^21^ versus 21 days p.i. in the current study. It is possible that these genes were also upregulated earlier on following *G*. *muris* infection, or that this response is more specific for *G*. *duodenalis* infection.

Furthermore, some downregulated transcripts that were identified in the study of Tako *et al*.^21^ and that were linked to enterocyte function and nutrient absorption, such as Amy2, Akp3, Try4 and Ctrb1, were not found in the current study. This could be due to similar reasons as the ones outlined for the discrepancy in mast cell-linked transcripts.

### Identification of the IL-17RA dependent transcriptional changes following a *G*. *muris* infection

As previously published, IL-17RA KO *G*. *muris* infected mice showed higher cumulative cyst counts than C57BL/6 WT *G*. *muris* infected mice. While C57BL/6 WT mice cleared the infection within a timeframe of 3 weeks, cyst excretion was still detectable in IL-17RA KO mice at 21 days p.i.^7^.

In order to identify the IL-17RA dependent intestinal responses following a *G*. *muris* infection, the transcriptome datasets obtained in C57BL/6 WT were subsequently compared to the datasets from C57BL/6 IL-17RA KO mice. In a first step, the datasets of the C57BL/6 IL-17RA KO infected mice and C57BL/6 IL-17RA KO uninfected control mice were analysed and compared to each other. Remarkably, while in C57BL/6 WT infected mice 844 genes were impacted by the infection, in C57BL/6 IL-17RA KO infected mice only 153 genes were differentially expressed compared to the C57BL/6 IL-17RA KO uninfected control mice, with 62 upregulated genes and 91 downregulated genes (Table S4). Pathway analysis on the complete set of DE genes revealed that functions related to apoptosis and damage were predicted to be increased, with important roles for IL-10 and Nos2 (Table S5). This effect is likely due to the prolonged presence of the parasite in the intestine, as it has been shown both *in vitro* ^28, 29^, as in human patients with chronic giardiasis^30^, that certain *Giardia* strains can induce apoptosis in enterocytes. The increased expression of the immune-regulatory cytokine IL-10 is likely important in the prevention of further tissue damage^31^. It is also interesting to note that Nos2 was transcriptionally upregulated following infection in the susceptible C57BL/6 IL-17RA KO mice. Although some studies have indicated that Nos2 was important in controlling a *Giardia* infection^32–34^, the data presented here supports the observations of Maloney *et al*.^35^ and Tako *et al*.^21^ that Nos2 did not play a crucial role in the protective immune response against *Giardia*.

**Figure 5:**
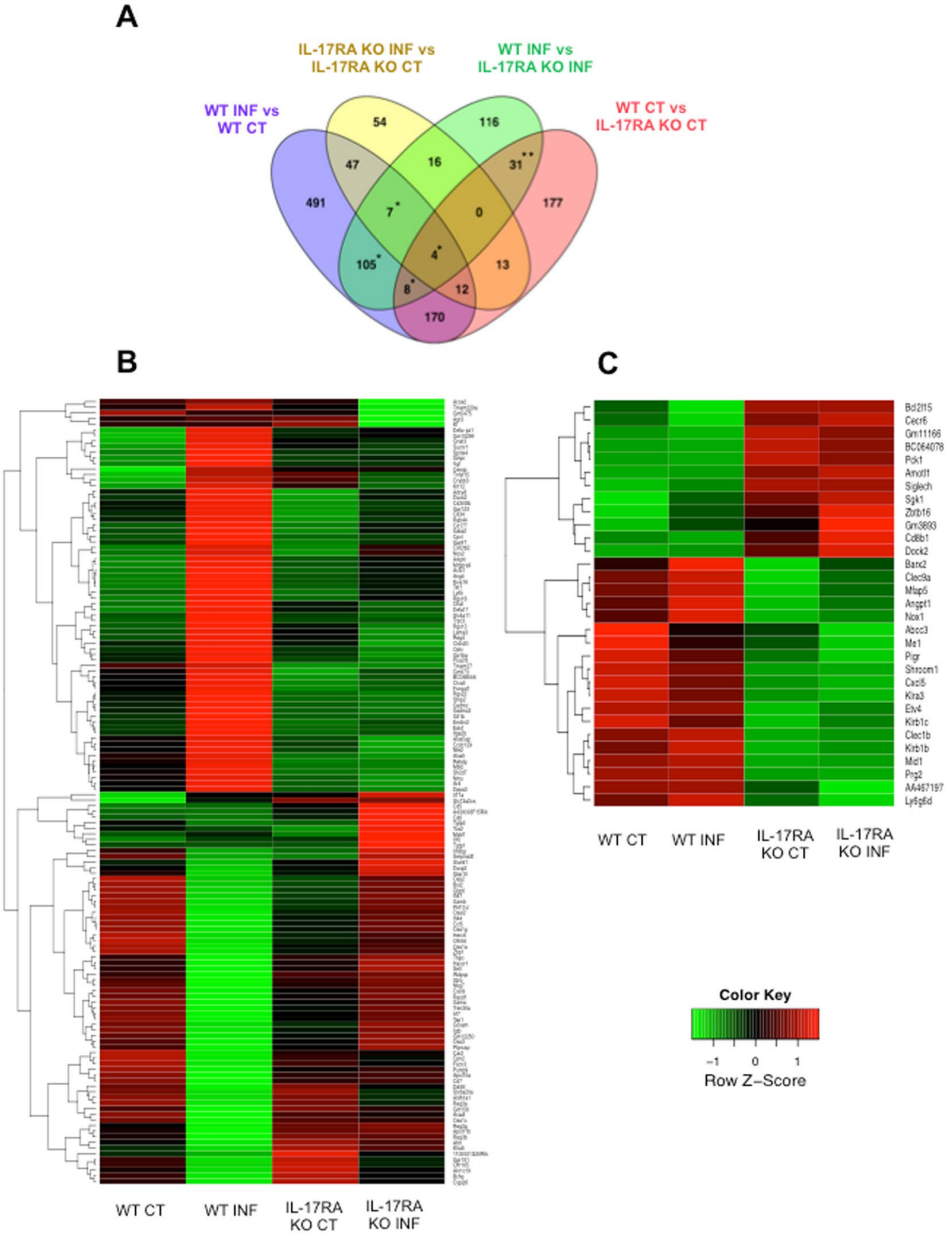
Differential expression analysis on the RNA-seq dataset was performed for 4 different comparisons between C57BL/6 WT and C57BL/6 IL-17RA KO infected and uninfected control mice. Venn diagrams and heatmaps that represent the different comparisons and subcategories were created. (**A**) Venn diagram representing differentially expressed genes in the RNA-seq dataset of C57BL/6 WT and C57BL/6 IL-17RA KO mice. Four different comparisons between WT and IL-17RA KO mice were further subdivided into 15 exhaustive subcategories. Subcategories marked with an *, contain genes that differentially respond to infection in WT infected mice versus IL-17RA KO infected mice, and in WT infected mice versus WT uninfected controls. The subcategory marked with **, contains genes that were differentially expressed between WT uninfected control versus IL-17RA KO uninfected control mice and between WT infected mice versus IL-17RA KO infected mice. (**B**). Heatmap of genes that differentially respond to infection in WT infected mice versus IL-17RA KO infected mice, and in WT infected mice versus WT uninfected controls (* in Fig. 5A). The color scale shows the relative expression patterns. (**C**) Heatmap of genes that were differentially expressed between WT uninfected control versus IL-17RA KO uninfected control mice and between WT infected mice versus IL-17RA KO infected mice (** in Fig. 5A).

By crosschecking the lists of DE genes for the different comparisons, graphically represented in Fig. 5A, 124 genes were identified (highlighted with an * in Fig. 5A and listed in Table S6), that responded differently following a *Giardia* infection in C57BL/6 WT mice compared to C57BL/6 IL-17RA KO mice, suggesting that their expression is regulated through IL-17RA. A heatmap showing the relative expression patterns of these genes in the different groups is shown in Fig. 5B and the 15 most upregulated genes in C57BL/6 WT mice compared to C57BL/6 IL-17RA KO mice are listed in Table 3.

**Table 3:** Top 15 most upregulated genes as identified by RNA-seq that differentially respond to infection in C57BL/6 WT infected mice versus C57BL/6 IL-17RA-KO infected mice (A) and also in WT infected mice versus WT uninfected controls (B).

Gene symbol	Gene name	RefSeq ID	Fold change A	Fold change B
TMEM27	Transmembrane protein 27	NM_020626	47,69	3,49
MBL2	Mannose-binding lectin 2	NM_010776	29,25	6,47
DPPA3	Developmental pluripotency-associated 3	NM_139218	13,09	2,97
NFE2	Nuclear factor, erythroid derived 2	NM_008685	11,45	2,86
GSDMC	Gasdermin C	NM_031378	9,33	3,90
GP1BA	Glycoprotein 1b, alpha polypeptide	NM_010326	8,61	3,52
RGS22	Regulator of G-protein signalling 22	NM_001195748	7,25	3,54
GSDMC2	Gasdermin C2	NM_177912	7,16	3,75
NMU	Neuromedin U	NM_019515	6,76	2,18
CCDC129	Coiled-coil domain containing 129	NM_001081665	5,94	2,54
GPR123	Adhesion G protein-coupled receptor A1	NM_177469	5,60	3,37
ANG4	Angiogenin, ribonuclease A family, member 4	NM_177544	5,56	7,20
DUOX2	Dual oxidase 2	NM_177610	5,55	4,10
RAB44	RAB44, member RAS oncogene family	NM_001002786	5,37	4,26
MRGPRA9	MAS-related GPR, member A9	NM_001288801	5,20	7,77

**Figure 6:**
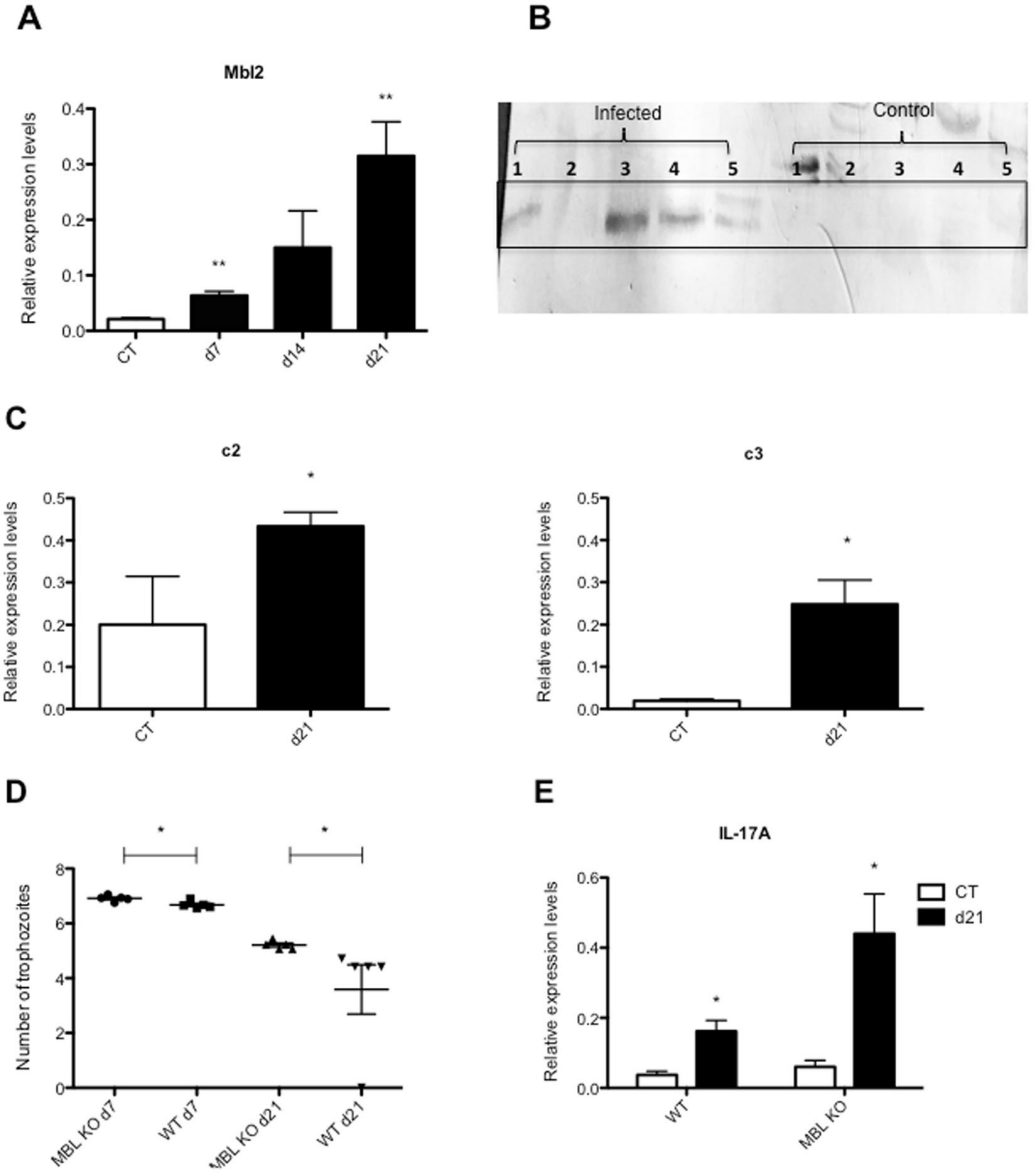
Mbl2 mRNA levels and protein levels and complement factors are upregulated following *G*. *muris* infection in C57BL/6 WT mice. Deficiency of Mbl has an impact on the course of a *G*. *muris* infection and on IL-17A mRNA levels. (**A**) Relative mRNA expression profile of Mbl2 in the small intestine of WT infected mice versus WT uninfected controls, as identified by qRT-PCR. mRNA levels in WT infected mice and WT uninfected control mice were estimated at day 7, day 14 and day 21 post infection (**p < 0.01). (**B**) Western blot with an anti-Mbl2 antibody on a water-insoluble extract of small intestinal tissue of WT infected (21 days p.i.) and WT uninfected control mice. Numbers 1 to 5 at the left represent 5 replicates of *G*. *muris* infected mice and at the right of uninfected control mice. A full-length uncropped blot is presented in Supplementary Figure 1. (**C**) Relative mRNA expression levels of the complement factors c2 and c3 in C57BL/6 WT infected mice were estimated by qRT-PCR at day 21 post infection, compared to C57BL/6 WT uninfected controls (*p < 0.05). (**D**) Total number of trophozoites present in the small intestine of infected WT and Mbl KO mice at day 7 and day 21 post infection (*p < 0.05). Values are represented on a logaritimic scale. (**E**) Relative mRNA expression levels of IL-17A in infected WT and Mbl KO mice were estimated by qRT-PCR at day 21 post infection and in WT and Mbl KO uninfected control mice (*p < 0.05, **p < 0.01).

Amongst the differentially expressed genes are several genes encoding antimicrobial proteins, such as defensin alpha pseudogene 1 (Defa-ps1), gasdermin C and C2 (Gsdmc/2), regenerating islet-derived family member 4 (Reg4) and resistin-like gamma (Retnlg), supporting previous observations that IL-17A can enhance the production of a range of antimicrobial proteins^36^. In addition, one of the most differentially expressed genes was mannose-binding lectin 2 (Mbl2) with almost 30-fold higher transcript levels in C57BL/6 WT mice compared to C57BL/6 IL-17RA KO mice. Mbl2 is a circulating C-type lectin that can activate the complement system upon binding carbohydrates with its carbohydrate recognition domains. Mbl2 has been shown to play a critical role in the innate immune defence against different pathogens^37, 38^. *In vitro* testing also revealed binding of Mbl2 to the surface of *G*. *duodenalis* trophozoites and proved its requirement in the complement-mediated killing of *G*. *duodenalis* trophozoites^39^. Furthermore, in a recent study, Li *et al*.^40^ showed that Mbl KO mice exhibited a delayed clearance of *G*. *duodenalis*. The data presented in the current study shows for the first time that the expression of Mbl2 is regulated through IL-17A and IL-17RA. Apart from the differential expression between the infected C57BL/6 WT and C57BL/6 IL-17RA KO mice, also the C57BL/6 WT uninfected control mice had higher Mbl2 transcript levels compared to C57BL/6 IL-17RA KO uninfected control mice (Table S7). Furthermore, the transcription profile of Mbl2 following a *G*. *muris* infection showed the same kinetics as IL-17A, with a significant upregulation 3 weeks post infection (Fig. 6A). This increased mRNA expression profile also coincided with higher levels of Mbl2 protein in the intestinal tissue of infected mice, as measured by Western blotting (Fig. 6B). A full-length uncropped blot is presented in Supplementary Figure 1. Once the lectin pathway is activated, it proceeds through the action of C2 and C4 to produce activated complement proteins further down the cascade, ultimately leading to the formation of the membrane attack complex and the chemotaxis and activation of inflammatory cells^41^. Importantly, in addition to Mbl2, several other components of the complement cascade, such as C7, C4bp, C1qtnf3 and C1qtnf9, as well as of mannose-binding lectin-associated serine protease 1 (Masp1), were also transcriptionally upregulated following a *G*. *muris* infection in C57BL/6 WT mice (Table S1). Furthermore, additional qRT-PCR analyses showed that also the core complement components C2 and C3 were significantly upregulated at day 21 post infection (Fig. 6C).

It has previously been shown that the activation of the complement cascade can induce the intestinal IL-17A axis through both C3aR^42^ as well as the complement anaphylatoxin C5a^43, 44^. In addition, Mbl2 can also modulate the release of both IL-1β and IL-6 production from monocytes, which could further trigger an IL-17 response^42, 43, 45^. It is therefore unclear whether the impaired ability of Mbl KO mice to clear a *Giardia* infection, as previously described (33), is due to the absence of a direct effect of Mbl2 on the trophozoites, or rather caused by a reduced IL-17A response in these mice. In order to investigate this, a *G*. *muris* infection study was performed in C57BL/6 Mbl KO mice to compare the IL-17A response to that of C57BL/6 WT mice. Similar as previously described^40^, trophozoite counts were significantly higher in C57BL/6 Mbl KO mice both at days 7 and 21 post infection compared to C57BL/6 WT mice (Fig. 6D). Analysis of the IL-17A expression levels by qRT-PCR showed that IL-17A was significantly upregulated in both the C57BL/6 WT and C57BL/6 Mbl KO mice at day 21 post infection. Interestingly, IL-17A levels were even higher in the C57BL/6 Mbl KO mice compared to C57BL/6 WT mice (Fig. 6E). This effect has previously also been reported in C57BL/6 Mbl KO mice that were subjected to intestinal inflammation by the administration of dextran sodium sulphate^45^. Based on this, the authors hypothesized that Mbl could act as an inhibitor to prevent an excessive inflammatory response. As an alternative explanation in the case of a *Giardia* infection, however, it is also possible that the higher number of trophozoites still present in the intestine of C57BL/6 Mbl KO mice, further triggers the IL-17A response. In contrast, Li *et al*. demonstrated that splenocytes collected from c3aR KO mice following *G*. *duodenalis* infection, produced reduced amounts of IL-17 *in vitro* in comparison to splenocytes from WT BALB/c mice. The authors suggest that these conflicting results could be attributed to the different species of mice that were used or the different timepoints that were analyzed^40^.

**Figure 7:**
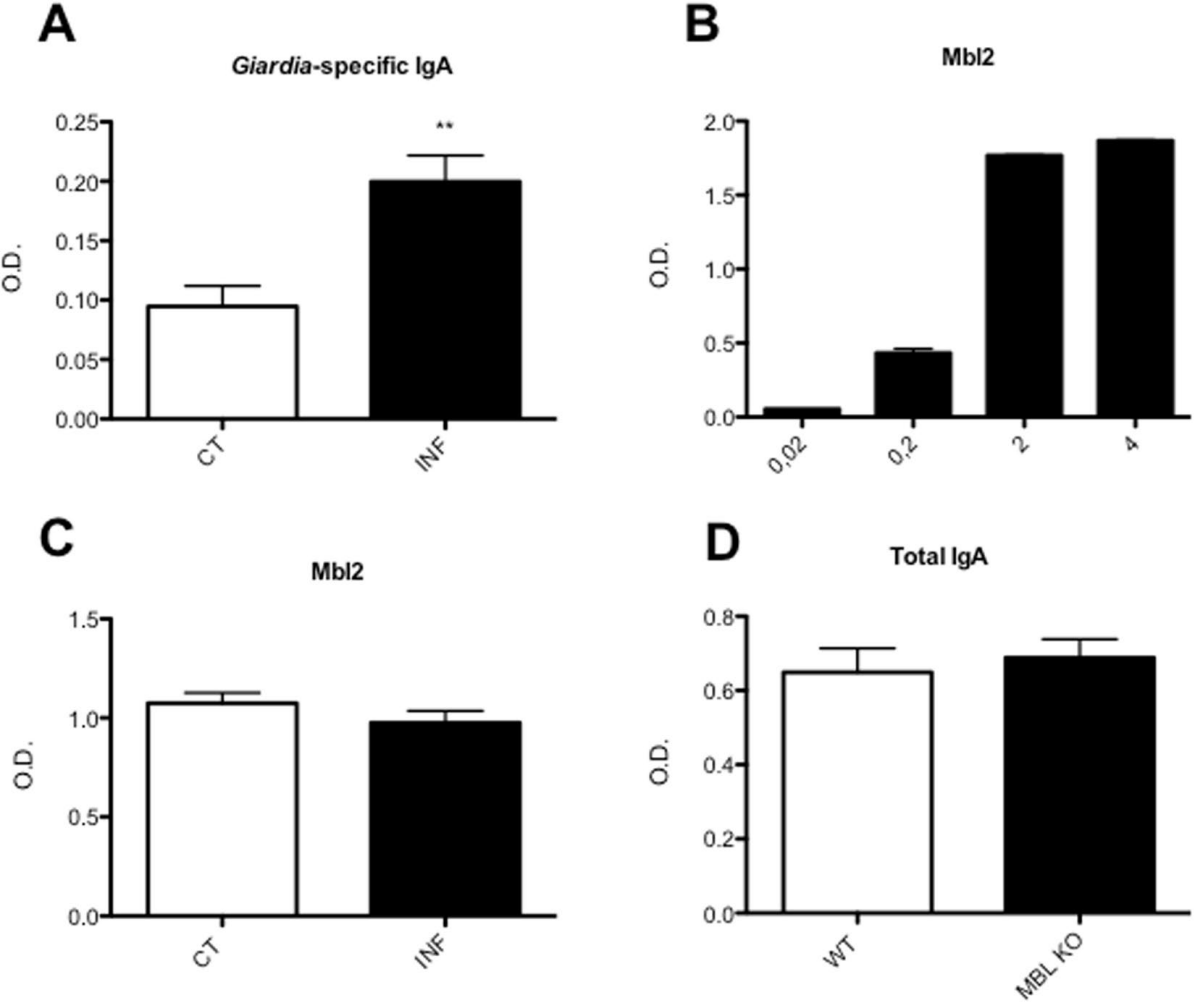
Fecal total IgA levels, *Giardia*-specific IgA levels, and Mbl2 levels, as measured by ELISA. (**A**) *Giardia*-specific IgA levels were measured in fecal samples from C57BL/6 WT uninfected control and *G*. *muris* infected mice. Optical density is shown. (**p < 0.01). (**B**) ELISA plates coated with trophozoites were incubated with recombinant Mbl2 in different concentrations (0.02–4 μg/mL). The amount of bound Mbl2 was measured through optical density. (**C**) ELISA plates coated with trophozoites were incubated with fecal samples from either C57BL/6 WT uninfected control mice or WT *G*. *muris* infected mice. Recombinant Mbl2 (2 μg/mL) was added and the amount of bound Mbl2 was measured. (**D**) Total fecal IgA levels were measured in C57BL/6 uninfected WT mice and Mbl KO uninfected mice.

In addition to Mbl2, IgA also has a protective role against *Giardia* infections, since mice deficient for IgA can not eradicate a *G*. *muris* infection^46^. Interestingly, previous research has shown that Mbl can actually bind to IgA and consequently activate the lectin pathway of the complement system^47^. In order to test this possibility in the context of a *Giardia* infection, first, we investigated the level of Mbl2 binding to trophozoites in the absence of parasite specific IgA’s. Plates were coated with *G*. *duodenalis* trophozoites and incubated with different concentrations of recombinant Mbl2. Mbl2 was shown to bind the trophozoites in a concentration-dependent manner. A concentration of at least 2 μg/ml of recombinant Mbl2 resulted in a saturated binding of the trophozoites (Fig. 7B). In a second phase, the effect of parasite specific IgA’s on Mbl2 binding was analysed. For this, the level of *Giardia*-specific IgA present in fecal samples from WT *G*. *muris* infected mice and WT uninfected control mice first needed to be determined. Hereto, 96-well plates were coated with *Giardia* trophozoites and incubated with fecal extracts. Samples from WT *G*. *muris* infected mice contained higher levels of *Giardia*-specific IgA that bound to the trophozoites, in comparison to WT uninfected control mice (Fig. 7A). Finally, plates coated with *Giardia* trophozoites were incubated with fecal samples from WT *G*. *muris* infected mice, containing *Giardia*-specific IgA, and from WT uninfected control mice and recombinant Mbl2 was subsequently added at 2 μg/ml. The presence or absence of bound IgA, originating from the fecal extracts, did not alter the amount of recombinant Mbl2 that bound to the trophozoites (Fig. 7C), suggesting that there was no binding between Mbl2 and IgA. This is in line with Terai *et al*. who reported that Mbl was not able to bind native IgA but only to aberrantly formed IgA that had abnormal glycosylation or denaturation^48^. In addition to the direct interaction between Mbl2 and IgA, we also investigated whether Mbl2 would affect IgA production and secretion. This was done by measuring total fecal IgA levels in WT and Mbl KO mice by ELISA. The results showed that there was no significant difference in total IgA levels between WT and Mbl KO mice (Fig. 7D), which is consistent with results shown by Li *et al*.^40^ in c3aR KO mice. This suggests that the diminished ability of Mbl KO mice to clear a *Giardia* infection is not due to reduced IgA levels.

In addition to the abovementioned cluster of genes that are transcriptionally regulated through IL-17RA following infection, there was a second cluster of 31 genes of which the expression was also IL-17RA dependent but independent on whether the animals were infected or not. These genes (highlighted with ** in Fig. 5A) are listed in Table 4 and their relative expression patterns are shown in Fig. 5C. A gene in this list that is extremely relevant in the context of a *Giardia* infection is the polymeric immunoglobulin receptor (pIgR). This receptor is responsible for the transport of IgA and IgM across epithelial tissues. Transport of IgA appears to be essential for the clearing of *G*. *muris*, since mice deficient for pIgR were unable to clear an infection with the parasite^49^. The transcriptome data presented here showed a 2-fold decrease in pIgR transcript levels in C57BL/6 IL-17RA KO mice compared to C57BL/6 WT mice. Quantitative RT-PCR analysis indicated that pIgR transcript levels were not affected by a *Giardia* infection (results not shown). It is also noteworthy that the gene encoding the J chain, which is a protein component of IgA and IgM that is required for their secretion into the mucosa, was also significantly upregulated following infection in C57BL/6 WT mice.

**Table 4:** List of upregulated genes as identified by RNA-seq in C57BL/6 WT uninfected control mice versus C57BL/6 IL-17RA KO uninfected control mice (A) and that also differentially respond upon infection in C57BL/6 WT infected mice versus C57BL/6 IL-17RA KO infected mice (B).

Gene symbol	Gene name	RefSeq ID	Fold change A	Fold change B
CLEC1B	C-type lectin domain family 1, member b	NM_019985	29,45	22,03
KLRB1B	Killer cell lectin-like receptor subfamily B member 1B	NM_030599	18,68	14,19
MID1	Midline 1	NM_183151	9,12	9,27
KLRB1C	Killer cell lectin-like receptor subfamily B member 1C	NM_008527	5,23	2,61
KLRA3	Killer cell lectin-like receptor, subfamily A, member 3	NM_010648	5,06	5,50
ETV4	Ets variant 4	NM_008815	4,05	3,02
CXCL5	Chemokine (C-X-C motif) ligand 5	NM_009141	3,38	3,18
NOX1	NADPH oxidase 1	NM_172203	3,36	3,99
MFAP5	Microfibrillar associated protein 5	NM_015776	2,82	2,32
CLEC9A	C-type lectin domain family 9, member a	NM_172732	2,75	2,42
ME1	Malic enzyme 1, NADP(+)-dependent, cytosolic	NM_008615	2,66	2,35
ABCC3	ATP-binding cassette, sub-family C, member 3	NM_029600	2,60	2,19
SHROOM1	Shroom family member 1	NM_027917	2,43	2,09
AA467197	Expressed sequence AA467197	NM_001004174	2,40	4,43
PRG2	Proteoglycan 2, bone marrow	NM_008920	2,38	2,44
BARX2	Barh-like homeobox 2	NM_013800	2,26	2,56
ANGPT1	Angiopoietin 1	NM_009640	2,25	2,43
PIGR	Polymeric immunoglobulin receptor	NM_011082	2,14	2,05
LY6G6D	Lymphocyte antigen 6 complex, locus G6D	NM_033478	2,03	3,91

## Conclusions

The outcome of this study provides further insights into the central role of the IL-17A/IL-17RA axis in orchestrating the protective immune response against *Giardia*. The upregulation of IL-17A and subsequent activation of IL-17RA does not only induce the production of an array of antimicrobial peptides and complement factors but also regulates the secretion of IgA into the intestinal lumen. The data presented here further indicate that the combination of complement activation through Mbl2 and parasite-specific IgA’s is crucial to combat a *Giardia* infection.

### Data availability statement

The RNAseq data is available upon request.

## Electronic supplementary material

Supplementary information

Table S1

Table S2

Table S3

Table S4

Table S5

Table S6

Table S7
