# Supplementary material for: Interleukin-17 receptor A (IL-17RA) as a central regulator of the protective immune response against Giardia
Source: Sci Rep. 2017 Aug 17;7:8520. doi: 10.1038/s41598-017-08590-x (PMC5561107; doi:10.1038/s41598-017-08590-x)
Supplement: Supplementary file 1 — Supplementary information [file 41598_2017_8590_MOESM1_ESM.pdf]

Interleukin-17 receptor A (IL-17RA) as a central regulator of the protective immune response against *Giardia*

Oonagh Paerewijck[1], Brecht Maertens[1], Leentje Dreesen[1], Frederik Van Meulder[1], Iris Peelaers[1], Dariusz Ratman[2], Robert W. Li[3], Erik Lubberts[4], Karolien De Bosscher[2], Peter Geldhof[1*]

[1]Department of Virology, Parasitology and Immunology, Laboratory of Parasitology, Faculty of Veterinary Medicine, Ghent University, Merelbeke, Belgium

[2]VIB Department of Medical Protein Research, Receptor Research laboratories, Nuclear Receptor Lab, Faculty of Medicine and Health Sciences, Ghent University, Ghent, Belgium

[3]United States Department of Agriculture, Agriculture Research Service, Animal Genomics and Improvement Laboratory, Beltsville, Maryland, United States of America

[4]Departments of Rheumatology and Immunology, Erasmus MC, University Medical Center, Rotterdam, The Netherlands.

[*]Corresponding author: Salisburylaan 133, 9820 Merelbeke, Belgium

Tel: +32 9 264 75 17

Fax: +32 9 264 74 96

E-mail: peter.geldhof@UGent.be

Supplementary Information

Supplementary Figure

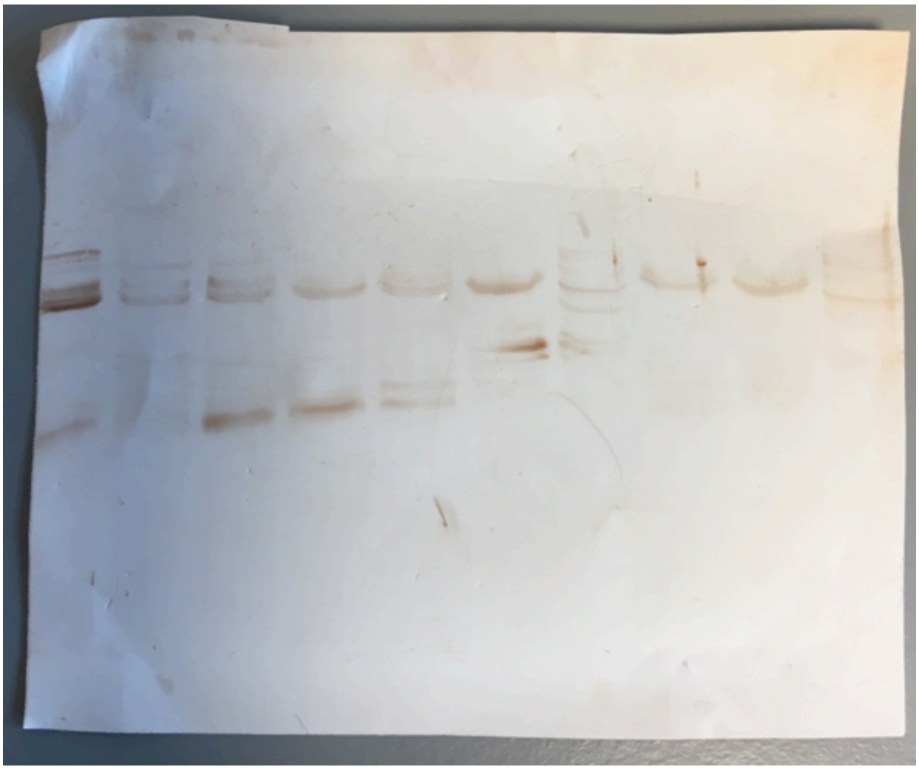

Figure S1. Full-length uncropped picture of a Western blot with an anti-Mbl2 antibody on a water-insoluble extract of small intestinal tissue of WT infected (21 days p.i.) and WT uninfected control mice.

Supplementary Files Legends

Table S1. List of the primer sequences used for qPCR analyses.

Table S2. Up- and down regulated genes in C57BL/6 WT infected mice versus C57BL/6 WT uninfected control mice.

Table S3. Pathway analysis of C57BL/6 WT infected mice versus C57BL/6 WT uninfected control mice.

Table S4. Upregulated and downregulated genes in C57BL/6 IL-17RA KO infected mice versus C57BL/6 IL-17RA KO uninfected controls.

Table S5. Pathway analysis of C57BL/6 IL-17RA KO infected mice versus C57BL/6 IL-17RA KO uninfected control mice.

Table S6. Upregulated and downregulated genes in C57BL/6 WT infected mice versus C57BL/6 IL-17RA KO infected mice.

Table S7. Upregulated and downregulated genes in C57BL/6 WT uninfected control mice versus C57BL/6 IL-17RA KO uninfected control mice.
